# Online Survey of the Impact of COVID-19 Risk and Cost Estimates on Worry and Health Behavior Compliance in Young Adults

**DOI:** 10.3389/fpubh.2021.612725

**Published:** 2021-03-29

**Authors:** Gabriella Imbriano, Emmett M. Larsen, Daniel M. Mackin, Akaisha Kaixuan An, Christian C. Luhmann, Aprajita Mohanty, Jingwen Jin

**Affiliations:** ^1^Neuroscience of Emotion, Cognition and Psychopathology Laboratory, Department of Psychology, Stony Brook University, Stony Brook, NY, United States; ^2^Klein Developmental Psychopathology Lab, Department of Psychology, Stony Brook University, Stony Brook, NY, United States; ^3^Cognition & Decision Making Lab, Department of Psychology, Stony Brook University, Stony Brook, NY, United States; ^4^Department of Psychology, The University of Hong Kong, Pokfulam, Hong Kong

**Keywords:** COVID-19, decision making, probability, cost, worry, health

## Abstract

The novel coronavirus COVID-19 pandemic is associated with elevated rates of anxiety and relatively lower compliance with public health guidelines in younger adults. To develop strategies for reducing anxiety and increasing adherence with health guidelines, it is important to understand the factors that contribute to anxiety and health compliance in the context of COVID-19. Earlier research has shown that greater perceived risk of negative events and their costs are associated with increased anxiety and compliance with health behaviors, but it is unclear what role they play in a novel pandemic surrounded by uncertainty. In the present study we measured (1) perceived risk as the self-reported probability of being infected and experiencing serious symptoms due to COVID-19 and (2) perceived cost as financial, real-world, physical, social, and emotional consequences of being infected with COVID-19. Worry was assessed using the Penn State Worry Questionnaire (PWSQ) and health compliance was measured as endorsement of the World Health Organization (WHO) health directives for COVID-19. Our results showed that greater perceived risk and costs of contracting the COVID-19 virus were associated with greater worry and while only costs were associated with greater compliance with health behaviors. Neither self-reported worry nor its interaction with cost estimates was associated with increased engagement in health behaviors. Our results provide important insight into decision making mechanisms involved in both increased anxiety and health compliance in COVID-19 and have implications for developing psychoeducational and psychotherapeutic strategies to target both domains.

## Introduction

The novel coronavirus COVID-19 pandemic poses a major public health threat in our current global community. The negative effects of this pandemic are experienced at the individual (e.g., insecurity and emotional isolation) and community (e.g., economic loss, school closures) level, and these effects translate into mental health consequences, such as anxiety and worry, as well as non-compliance with public health directives, such as social distancing and hand washing (1). The United Nations has described the coronavirus pandemic as having “the seeds of a major mental health crisis” (2), with evidence of increased anxiety and depression in the general population (3–5), especially for young adults (6). For effective prevention and treatment of anxiety, including screening, psychoeducation and psychological therapy, it is important to understand factors that contribute to COVID-19-related anxiety in young adults.

Additionally, while the present public health response to the COVID-19 pandemic relies primarily on behavioral directives, non-compliance to these directives has been noted due to a variety of reasons (7–9), especially in younger adults (10). For effective messaging and psychoeducation, it is important to understand the decision-making mechanisms that contribute to compliance with COVID-19-related public health directives critical to effective containment of viral spreading in young adults. Hence, in the present study, we focused on two key aspects of decision making: perceived risk or probability of experiencing negative events and the perceived cost (consequences) of these negative events. Specifically, we examined whether perceived risk and costs of developing COVID-19 influences levels of worry and compliance to health recommendations in young adults.

There is considerable evidence suggesting that anxiety can be driven by decision making biases that involve exaggerated estimates of the probability of negative events, and exaggerated estimates of the cost (i.e., consequences) associated with the negative events (11–13). Overestimation of feared consequences is a common transdiagnostic feature of anxious pathology (14). Prior research has shown that inflation of perceived risk of encountering negative situations more generally is related to increased trait anxiety (15). Clinical populations with generalized anxiety disorder, which is characterized by high levels of worry, also perceive negative events as being more probable [for review, (16, 17)]. Additionally, Berenbaum et al. (18) showed that greater self-reported worry was associated with higher perceived probability of negative outcomes as well as more severe cost estimates of these negative outcomes in a sample of college students. However, anxiety disorders or symptoms are not uniformly characterized by overestimation of probabilities and costs of negative or feared outcomes. Instead, anxiety may exhibit specific relationships with risks and costs (19, 20). Considering the higher rates of anxiety observed in younger adult populations during the COVID-19 pandemic (6), it is important to assess perceived risks and costs of COVID-19 infection to examine if these factors contribute uniquely to increases in anxiety symptoms such as worry severity in young adults during the pandemic.

While the COVID-19 pandemic is characterized by novelty and uncertainty, it is clear that health-related decisions such as socially distancing oneself, washing hands frequently, not touching one's face, and avoiding non-essential travel will be effective in reducing the spread of COVID-19 (21). However, while factual information about health threats and methods of risk-reduction is necessary, it is often not sufficient for compliance. Research in decision making shows that effective human behavior is guided by accurate assessments of the risk with which negative events will occur and the cost associated with those negative events (22–27). Indeed, most decision-oriented theories of health behavior propose that the judgments of the probability of future harm are key to whether preventive action is taken (28, 29). For example, prior investigations have shown that adherence to vaccine protocols was significantly predicted by estimated probabilities of getting infected with influenza as well as costs associated with influenza (30). Additionally, evidence suggests that self-reported judgements of how vulnerable someone feels regarding their health can have more of an impact on subsequent behavior than judgements based on statistical evidence (29, 31, 32). Since younger adults tend to show lower compliance to COVID-19-related health directives (10, 33), perceived risk and estimates of COVID-19 infection may offer clues about this non-compliance.

Finally, more recent models of health-related risk perception and decision making have differentiated more deliberative components of decision making from the more affective components (29, 34–36). Worry or anxiety about a threat is considered to be an affective analog to deliberative risk perceptions (35). Additionally, it has been proposed that more complex interplay between deliberative and affective factors can influence health-related behaviors (29). For example, when deliberative and affective perceptions converge, the potential for action is the greatest (29). This suggests the need to examine the contribution of anxiety and its interaction with risk and cost estimates with COVID-19 health behavior compliance in young adults.

In sum, prior research on health behaviors has demonstrated the importance of risks and cost estimates in increased anxiety and compliance with health recommendations. However, these factors have not yet been examined in the context of an ongoing pandemic such as COVID-19. Additionally, this is particularly important to assess in young adults who can be powerful vectors for disease transmission as they are more likely to be asymptomatic carriers of COVID-19 (37–39) and show increased anxiety and decreased health compliance. Hence, in the present study we aimed to examine the differential association of perceived risks and costs of COVID-19 related outcomes with symptoms of anxiety, particularly worry, measured as a dimensional variable, as well as compliance with health-related behaviors in a sample of college students in the New York metro area. Perceived risks were measured as the self-reported probability of being infected by COVID-19 and experiencing serious symptoms due to COVID-19, while perceived cost was measured as financial, real-world, social, and emotional consequences of being infected with COVID-19. We hypothesized that greater perceived risks and costs of contracting COVID-19 would be associated with greater worry and greater engagement with health behaviors. Furthermore, we hypothesized that greater costs of contracting COVID-19, greater worry, and their interaction would be associated with greater compliance with health directives for reducing the spread of COVID-19.

## Method

### Participants

The data were collected from 261 undergraduate students at Stony Brook University (SBU) who participated in this experiment for partial course credit. Participant demographics for the final sample can be seen in Table 1. Because the experiment was hosted online via Sona Systems for SBU, potential participants needed to be enrolled at Stony Brook University from April 21, 2020 to May 8, 2020 and 18 years of age or older to be eligible for participation. Participants were excluded from analyses for completing the questionnaires in <10 min (*N* = 21) due to suspected inattentiveness, leaving a final sample of 240 participants. The average time to complete the study for the final sample of participants was 27.3 min. Participants consented to participation prior to engaging in any procedural tasks. This investigation was approved by the Stony Brook University Institutional Review Board.

**Table 1:** Participant demographics.

	***N***	**%**
Gender	—	—
Female	150	62.5
Male	90	37.5
Race	—	—
White/Caucasian	73	30.4
Hispanic/Latino	37	15.4
Black	18	7.5
Asian/Pacific Islander	101	42.1
Other	11	4.6
		Mean (SD)
Age (years)		19.9 (2.4)

### Measures

#### COVID-19 Health Behaviors

Health behavior adherence was assessed based on recommendations appearing on the website of the World Health Organization (40) during the first week of April 2020. A total of 8 items were administered pertaining to changes in frequency of the following health behaviors: handwashing; use of hand sanitizer; social distancing; avoiding touching eyes, nose and mouth; covering mouth and nose with bent elbow or a tissue when coughing or sneezing; disinfecting or washing surfaces that are frequently touched. All questions were framed in terms of the respondent's behavior in the past week. Responses ranged on a 4-point scale from Not at all to Very much increased. Individual item responses were averaged into a composite summary score. The set of health behavior questions displayed acceptable internal consistency (Cronbach's *a* = 0.71, Table 2), supporting the use of an average score as a unitary index.

**Table 2:** Descriptive statistics for measures (*n* = 240).

	**Factor**	**μ**	**σ**	***SK***	***Rku***	**α**
**MEASURE**						
1	COVID-19 Health Behavior	17.93	4.01	0.06	−0.60	0.71
2	COVID-19 Costs	103.31	23.21	−0.60	0.38	0.95
3	Risk of Contracting COVID-19	3.49	1.34	0.26	0.01	–
4	Risk of Becoming Seriously Ill due to COVID-19	2.88	1.34	0.34	−0.54	–
5	PSWQ	53.25	13.36	0.04	−0.63	0.83

*μ, mean; σ, standard deviation; SK, skewness; Rku, kurtosis; α, Cronbach's Alpha*.

#### COVID-19 Perceived Risks

Participants rated perceived risks of two COVID-19 related outcomes. The first outcome was the risk of being infected with COVID-19. The item read, “*I think my chances of getting infected with COVID-19 are.”* The second outcome was the risk of becoming seriously ill due to COVID-19. The item read, “*I think my chances of becoming seriously ill due to COVID-19 are.”* Participants responded to each of these items on a 7-point scale with scores ranging from *1* (*Almost zero) to 7 (Almost certain)*. Wording of the risk estimate items were adapted from prior research investigating the perception of influenza risks (31).

#### COVID-19 Perceived Cost

Projected consequences of COVID-19 infection were measured using a novel, 30-item measure. Five domains of projected illness costs were assessed, including perceived physical symptom consequences (“*If I get infected with COVID-19, I will have breathing problems*”; responses ranged from *1*-*Not distressing* to *5-Severely distressing)*; daily functioning consequences (“*If I get infected with COVID-19, it will impact my ability to do my work*”; responses ranged from *1*-*Not at all* to *5-Severely*); emotional/mood consequences (“*If I get infected with COVID-19, I will feel irritable*”; responses ranged from *1*-*None* to *5-Severe*); social consequences (“*If I get infected with COVID-19, I will impact people by making them worry*”; responses ranged from *1*-*Not at all* to *5-Severely*); and financial/livelihood consequences (“*If I get infected with COVID-19, I will suffer financially*”; responses ranged from *1*-*Not at all* to *5-Severely*). The 30-item inventory displayed excellent internal consistency (Cronbach's *a* = 0.95, Table 2). Individual item responses were averaged into a summary score.

#### Worry

Worry was dimensionally assessed using the Penn State Worry Questionnaire [PSWQ; (41)], a 16-item self-report measure that assesses symptoms of worry by asking participants to rate on a 1 (“*Not at all typical of me*”) to 5 (“*Very typical of me*”) scale how characteristic various worry related statements are for them (for e.g., “*My worries overwhelm me.”*). The PSWQ has demonstrated high reliability (*r* = 0.93) and internal consistency (Cronbach's *a* = 0.83, Table 2) as a measure of worry. Individual item responses were averaged into a summary score.

### Research Procedure

Participants completed the study by logging into the Sona Systems website for SBU students. This system is used for students to sign up to participate in studies for course credit at SBU. If they elected to participate in the study, they clicked a link that allowed them to complete the study via Qualtrics survey software. First, participants provided consent. Order of questionnaire administration was randomized with the exception of demographic questions, which were presented first. Following completion of the survey, participants were automatically directed to a debriefing form. Data for this manuscript was obtained from a subset of questionnaires (see Appendix) given for a larger study which included 12 total questionnaires [for description of other data that were collected, see (42)].

### Data Analytic Strategy

First, Pearson's correlations were estimated between continuous variables to examine whether risk of contracting COVID-19, risk of becoming seriously ill due to COVID-19, and COVID-19 perceived costs were related to worry and COVID-19 health behaviors. All correlations were corrected for multiple comparisons at the 0.05 level and were thus assessed as significant if they exceeded the *p* < 0.007 threshold for corrected significance.

Next, a path model with three independent variables (perceived risk of contracting COVID-19, perceived risk of becoming seriously ill from COVID-19, and perceived costs of COVID-19) and two correlated dependent variables (worry and COVID-19 health behaviors) was constructed. Participant age and gender were included as covariates to account for potential effects demonstrated in prior investigations (43). This allows for examination of the unique effects of each independent variable on the unique variance of each dependent variable, beyond the effect of the other independent variables and the covariates. Because this model is just identified, model fit diagnostics are not available.

Finally, because worry and its interaction with COVID-19 perceived risk and or cost estimates may have independent effects on adherence to health behaviors beyond the contribution of main effects independently, we conducted a hierarchical linear regression analysis to examine whether the interactive effects of COVID-19 perceived risks and or costs with worry contributed additional variance in prediction of COVID-19 health related behaviors. Again, participant age and gender were included as covariates. All independent variables were mean centered before calculating interaction terms.

Correlation and hierarchical regression analyses were conducted using IBM SPSS Statistics (version 26; IBM, Armonk, N.Y.). The path model was estimated in Mplus 8 [version 8.3; (44)] using a maximum likelihood estimator, which is appropriate for continuous variables (45).

## Results

### Bivariate Correlations

Correlational analyses showed that greater perceived risk of contracting COVID-19, *r*_(238)_ = 0.211, *p* = 0.001, experiencing more serious COVID-19 symptoms, *r*_(238)_ = 0.244, *p* < 0.001, and greater COVID-19 perceived costs, *r*_(238)_ = 0.339, *p* < 0.001, were associated with greater severity of worry symptoms. Additionally, beliefs about COVID-19 perceived costs were positively associated with engagement in health behaviors, *r*_(238)_ = 0.199, *p* = 0.002, such that the greater one's estimation of the negative costs of COVID-19, the more likely respondents were to endorse an increased engagement with preventative health behaviors. However, perceived risk of contracting COVID-19 was not related to COVID-19 health behaviors, *r*_(238)_ = 0.108, *p* = 0.096, nor was the perceived risk of becoming seriously ill due to COVID-19, *r*_(238)_ = 0.048, *p* = 0.455.

### Path Model Predicting Worry and Health Behaviors

**Figure 1:**
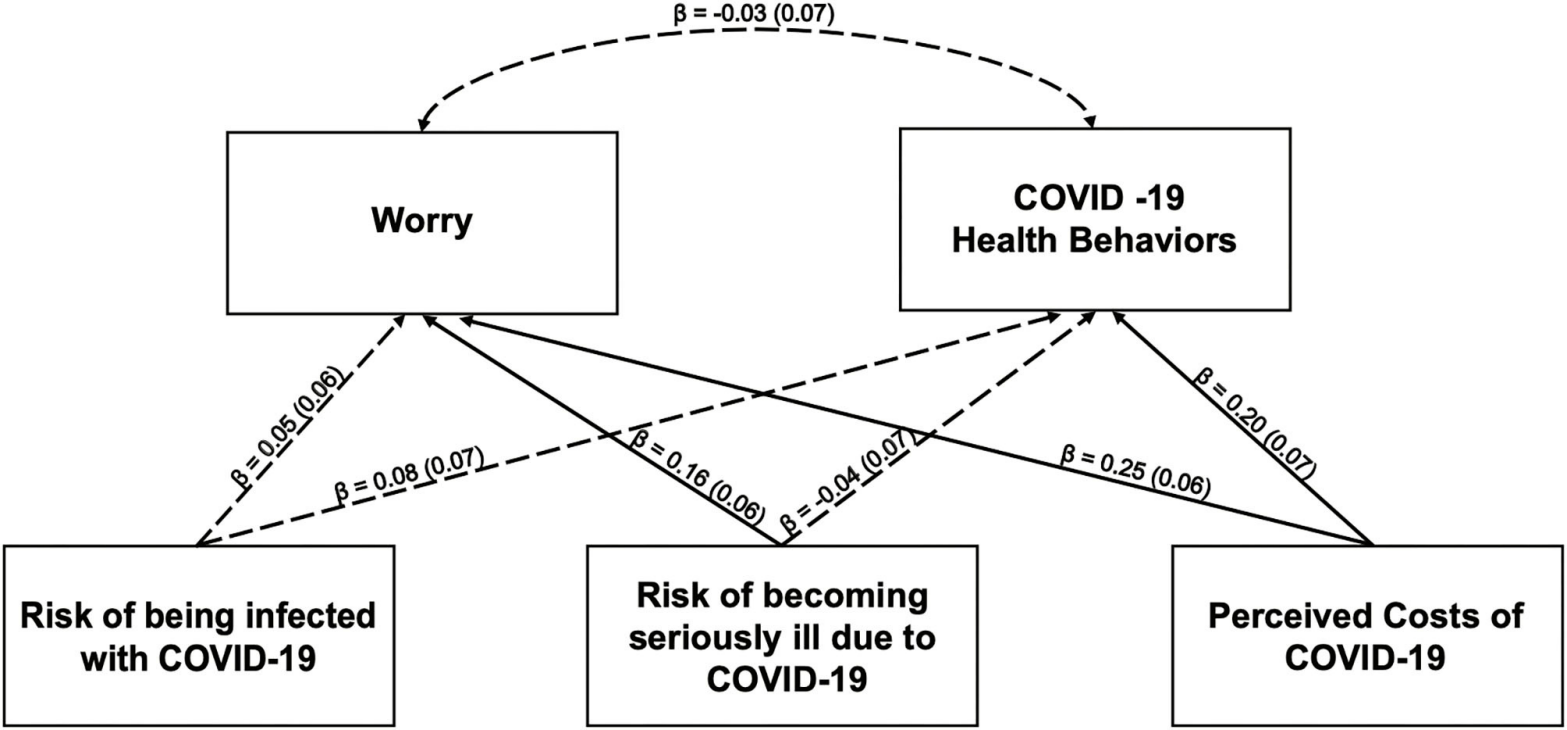
Path model estimating the impact of the risk of being infected with COVID-19, the risk of becoming seriously ill due to COVID-19 and costs of COVID-19 on anxiety and health behaviors. All path estimates are standardized to allow for comparison of magnitude between paths. Non-significant paths are indicated with dashed lines. Significant paths are indicated with solid lines. Gender and age were included as covaries but omitted for the figure for simplicity. Path estimates for the covariates are as follows: COVID-19 Health Behaviors on Gender (*β* = −0.01, SE *β* = 0.06, *p* = 0.82; COVID-19 Health Behaviors on Age (*β* = 0.12, SE *β* = 0.06, *p* = 0.06); Worry on gender (*β* = −0.29, SE *β* = 0.06, *p* ≤ 0.001); Worry on Age (*β* = −0.07, SE *β* = 0.06, *p* = 0.25).

Results from the path model with perceived risk of contracting COVID-19, perceived risk of becoming seriously ill from COVID-19, and COVID-19 perceived costs predicting worry and COVID-19 health behaviors are shown in Figure 1. After accounting for all other predictors and covariates, only the perceived costs of COVID-19 predicted COVID-19 health behaviors, such that greater levels of perceived costs were associated with significantly greater levels of health behavior change. Overall, these variables accounted for a significant proportion of variance in health behavior change (*R*^2^ = 0.06*, p* < 0.05). When examining the influence of the independent variables and covariates on level of worry, higher perceived risk of becoming ill, higher perceived costs of having COVID-19, and being female were all uniquely and significantly associated with greater levels of worry. Overall, these variables accounted for a significant proportion of variance in worry (*R*^2^ = 0.24, *p* < 0.001).

### Moderation Model Predicting Health Behaviors

Findings from the hierarchical regression analysis examining the main and interactive effects of COVID-19 perceived costs, worry and their interaction predicting COVID-19 health behavior controlling for age and gender showed that neither the main effect of worry nor its interaction with COVID-19 perceived costs accounted for significant additional variance in COVID-19 related health behaviors, although the predictors did account for a significant proportion of variance in the dependent variable overall, *R*^2^ = 0.05, *Adjusted R squared* = 0.03, *F*_(5,234)_ = 2.68, *p* = 0.022. COVID-19 perceived costs remained a significant predictor of COVID-19 health behavior controlling for all other variables, *b* = 0.22, *p* < 0.01.

## Discussion

Almost a third of Americans have reported increased anxiety during the peak of the COVID-19 pandemic, with nearly 50% of young adults reporting anxiety increases (6). While public health agencies have recommended several non-pharmacological health behaviors that are effective in reducing transmission rates and risk of illness (21), behavioral responses to COVID-19 have been mixed with evidence of non-compliance, especially in young adults (6). Our study sought to understand the factors that contribute to this increased anxiety and decreased compliance with health directives in young adults. Our results showed that higher perceived risk of becoming ill and higher perceived costs of COVID-19 infection was associated with increased worry severity. Only negative consequences and costs one would incur from contracting the virus were uniquely associated with increased preventive health behaviors. However, neither greater self-reported worry nor its interaction with cost estimates of COVID-19 infection were related to increased engagement in health behaviors.

Worry can serve to be more harmful than helpful in the case of the COVID-19 pandemic; hence, global health advocates may want to consider integrating worry assessment and reduction when educating the public on current challenges they may be facing. Our findings suggest that an important contributing factor to increases in worry is the perception that one might incur several negative costs or consequences as a result of contracting COVID-19. Investigators are now beginning to uncover the economic toll of COVID-19 through productivity losses (46), specifically with regards to the cost of unmet mental health needs during the pandemic (47), which bolsters the importance of disseminating mental health interventions where possible. Targeting catastrophizing has been an integral part of cognitive-behavioral interventions for worry (48) and maybe effective in reducing worry at the time of the current pandemic. It is important to note that in interventions such as cognitive restructuring for worry, possible negative consequences or costs of outcomes are not invalidated or minimized. Rather, the focus of treatment is shifted to agency in actions that would productively decrease an individual's risk of the feared outcome, for example the health behaviors recommended for COVID-19. Also, particularly relevant for young adult populations, it may be important to examine how social media and news consumption contributes to increased cost estimates because recent work from Gao et al. (49) shows that social media use during the COVID-19 outbreak in China was related to increased anxiety and depression symptoms.

A great deal of research has focused on understanding the psychological determinants of health behaviors [e.g., (50)]. Models of health behavior include a number of common determinants including intentions, self-efficacy, treatment outcome expectancies, perceived risk or susceptibility and perceived severity, that contribute differentially to different health problems (50). According to the predominant health belief model (51), the importance of probability and cost estimates relies on the fact that when the disease threat is viewed as low probability and its consequences mild, there is a low motivation to engage in health behavior change and individuals ignore health directives (51). Our results show that it is not perceived probability but perceived costs of contracting COVID-19 illness that predicts health compliance. The relatively low importance of perceived probability in case of COVID-19 pandemic may be because pandemics tend to be low probability but high consequence events, perhaps giving individuals less opportunity to factor probability estimates in their decision making. Furthermore, the novelty of this pandemic with constantly changing information maybe reducing reliance on probability estimates in a situation where the emotional, social, financial, and physical consequences are being experienced. Finally, our results diverge from another study that found an association between probability estimates and health behavior (52) however, this study did not measure cost estimates allowing participants to separate the two in their responses and did not focus specifically on younger adults who tend to underestimate risk of diseases (53, 54). Given the importance of cost estimates in the present study, it is important to consider perceived costs in psychoeducation and messaging for encouraging health-related behaviors in young adults.

This investigation was limited by the availability of COVID-19-related assessment measures at the time of data collection. However, the scales used above demonstrated internal consistency. Additionally, at the time of data collection and approval, wearing masks was not unanimously regarded as an accepted practice for mitigating viral spread in the United States so we were unable to collect data related to that aspect of prevention. However, future iterations of this paradigm will include mask-related questions. An additional limitation of this study was the lack of generalizability given the nature of our sample. The population of this study was composed of college-age students enrolled in a New York Metro area university from April 21, 2020 to May 8, 2020. Therefore, the results should be interpreted with respect to the specificity of this group, as the geographic and temporal specificity of data collection is germane to the investigation of the trajectory of COVID-19 related beliefs. Additionally, the brevity of the administration window did constrain the magnitude of our sample size and the range of attitudes toward COVID-19 that may have evolved over time. Particularly relevant is that toward the beginning of the COVID-19 outbreak, younger individuals were considered to be less vulnerable than older populations to severe health outcomes (55). The results presented here, while not nationally representative, do offer important insights into mental health and compliance with health behaviors in a population demographic that have become of greater interest as we learn more about asymptomatic viral spreading. To expand on the current results, future studies may seek to investigate other factors that contribute to positive health behavior engagement such as consistent exposure to accurate and comprehendible scientific information and guidance.

## Conclusion

Young adult populations have garnered attention throughout the COVID-19 pandemic, because they have the potential to be vectors for viral transmission, are especially prone to anxiety, and show relatively lower compliance with public health guidelines. However, the factors contributing to increases in anxiety and health-related behavioral decision-making have not been well-examined. The current investigation shows that in a limited sample of New York area college students, greater perceived risk of falling seriously ill due to COVID-19 and the costs of contracting it were associated with increased worry severity while only perceived costs were associated with increased engagement in CDC advised health behaviors. These findings clarify the decision making mechanisms involved in both increased anxiety and health compliance in COVID-19.

## Data Availability Statement

The dataset presented in this study can be found in online repositories. Data used for this project is available online at Open Science Framework (OSF; https://osf.io/cgzsd/).

## Ethics Statement

The studies involving human participants were reviewed and approved by Stony Brook University Institutional Review Board. The patients/participants provided their written informed consent to participate in this study.

## Author Contributions

AM supervised the project. JJ, EL, and AM conceived of the experiment and compiled the materials for the experimental design. AM, CL, and JJ advised on theoretical background and analysis considerations. EL collected the data. JJ, GI, and DM analyzed the data. GI, AM, and AA wrote the manuscript with support from JJ, EL, and DM. All authors helped shape the manuscript and provided essential feedback to finalize the completed manuscript.

## Conflict of Interest

The authors declare that the research was conducted in the absence of any commercial or financial relationships that could be construed as a potential conflict of interest.
