# Supplementary material for: Online Survey of the Impact of COVID-19 Risk and Cost Estimates on Worry and Health Behavior Compliance in Young Adults
Source: Front Public Health. 2021 Mar 29;9:612725. doi: 10.3389/fpubh.2021.612725 (PMC8039118; doi:10.3389/fpubh.2021.612725)
Supplement: Supplementary file 1 [file Data_Sheet_1.docx]

Appendix

**COVID-19 Health behaviors**

**Instructions:** Please answer each of the following questions related to your behaviors *OVER THE PAST WEEK (7 DAYS).*

*In the past week...*

1. On average, how many times PER DAY have you washed your hands? (0-1, 2-4, 5-7, 7+)
   1. To what extent is this an increase in comparison to your normal behavior? (Not at all, Slightly increased, Moderately increased, Very much increased)
2. On average, how many times PER DAY have you used hand sanitizer? (0-1, 2-4, 5-7, 7+)
   1. To what extent is this an increase in comparison to your normal behavior? (Not at all, Slightly increased, Moderately increased, Very much increased)

*In the past week, in comparison to your normal behavior before the current situation with COVID-19, to what extent have you...*

1. ...changed your normal behavior to engage in “social distancing” (eg. refraining from attending gatherings, using public transit or being in public areas)?

(Not at all, Slightly increased, Moderately increased, Very much increased)

1. ...increased your attempts to avoid touching your eyes, mouth and nose?

(Not at all, Slightly increased, Moderately increased, Very much increased)

1. ...increased your attempts to cover your mouth and nose with bent elbow or a tissue when coughing or sneezing?

(Not at all, Slightly increased, Moderately increased, Very much increased)

1. ...increased your instances of disinfecting or washing surfaces that are frequently touched (such as tables, doorknobs, light switches, countertops)?

(Not at all, Slightly increased, Moderately increased, Very much increased)

**COVID-19 Perceived Risks**

| 1 | I think my chances of getting infected with COVID-19 are: | Almost zero  1 | Very small  2 | Small  3 | Moderate  4 | Large  5 | Very large  6 | Almost certain  7 |
| --- | --- | --- | --- | --- | --- | --- | --- | --- |
| 2 | I think my chances of becoming seriously ill due to COVID-19 are | Almost zero  1 | Very small  2 | Small  3 | Moderate  4 | Large  5 | Very large  6 | Almost certain  7 |

**COVID-19 Perceived Cost**

| 1 | If I get infected with COVID-19 I will have the following symptoms |  |  |  |  |  |
| --- | --- | --- | --- | --- | --- | --- |
|  | Headache | Not distressing  1 | Very mildly distressing  2 | Mildly distressing  3 | Moderately distressing  4 | Severely distressing  5 |
|  | Fever | Not distressing  1 | Very mildly distressing  2 | Mildly distressing  3 | Moderately distressing  4 | Severely distressing  5 |
|  | Body Ache | Not distressing  1 | Very mildly distressing  2 | Mildly distressing  3 | Moderately distressing  4 | Severely distressing  5 |
|  | Fatigue | Not distressing  1 | Very mildly distressing  2 | Mildly distressing  3 | Moderately distressing  4 | Severely distressing  5 |
|  | Neck Pain | Not distressing  1 | Very mildly distressing  2 | Mildly distressing  3 | Moderately distressing  4 | Severely distressing  5 |
|  | Loss of sleep | Not distressing  1 | Very mildly distressing  2 | Mildly distressing  3 | Moderately distressing  4 | Severely distressing  5 |
|  | Loss of Appetite | Not distressing  1 | Very mildly distressing  2 | Mildly distressing  3 | Moderately distressing  4 | Severely distressing  5 |
|  | Cough | Not distressing  1 | Very mildly distressing  2 | Mildly distressing  3 | Moderately distressing  4 | Severely distressing  5 |
|  | Sore Throat | Not distressing  1 | Very mildly distressing  2 | Mildly distressing  3 | Moderately distressing  4 | Severely distressing  5 |
|  | Nasal Congestion | Not distressing  1 | Very mildly distressing  2 | Mildly distressing  3 | Moderately distressing  4 | Severely distressing  5 |
|  | Breathing Problems | Not distressing  1 | Very mildly distressing  2 | Mildly distressing  3 | Moderately distressing  4 | Severely distressing  5 |
| 2 | If I get infected with COVID-19, it will impact my ability to |  |  |  |  |  |
|  | Get out of bed | Not at all  1 | Very mildly  2 | Mildly  3 | Moderately  4 | Severely  5 |
|  | Prepare meals | Not at all  1 | Very mildly  2 | Mildly  3 | Moderately  4 | Severely  5 |
|  | Perform daily routines | Not at all  1 | Very mildly  2 | Mildly  3 | Moderately  4 | Severely  5 |
|  | Leave home | Not at all  1 | Very mildly  2 | Mildly  3 | Moderately  4 | Severely  5 |
|  | Concentrate on work | Not at all  1 | Very mildly  2 | Mildly  3 | Moderately  4 | Severely  5 |
|  | Complete my work | Not at all  1 | Very mildly  2 | Mildly  3 | Moderately  4 | Severely  5 |
| 3 | If I get infected with COVID-19, I will feel |  |  |  |  |  |
|  | Irritable | None  1 | Very mild  2 | Mild  3 | Moderate  4 | Severe  5 |
|  | Helpless | None  1 | Very mild  2 | Mild  3 | Moderate  4 | Severe  5 |
|  | Worried | None  1 | Very mild  2 | Mild  3 | Moderate  4 | Severe  5 |
|  | Frustrated | None  1 | Very mild  2 | Mild  3 | Moderate  4 | Severe  5 |
|  | If I get infected with COVID-19, I will impact people by |  |  |  |  |  |
|  | Making them worry | Not at all  1 | Very mildly  2 | Mildly  3 | Moderately  4 | Severely  5 |
|  | Being a burden | Not at all  1 | Very mildly  2 | Mildly  3 | Moderately  4 | Severely  5 |
|  | Limiting their lives | Not at all  1 | Very mildly  2 | Mildly  3 | Moderately  4 | Severely  5 |
|  | Needing to depend on them | Not at all  1 | Very mildly  2 | Mildly  3 | Moderately  4 | Severely  5 |
|  | Not being able to take care of people dependent on me | Not at all  1 | Very mildly  2 | Mildly  3 | Moderately  4 | Severely  5 |
|  | Spreading infection to them | Not at all  1 | Very mildly  2 | Mildly  3 | Moderately  4 | Severely  5 |
| 4 | If I get infected with COVID-19, I will |  |  |  |  |  |
|  | Suffer financially | Not at all  1 | Very mildly  2 | Mildly  3 | Moderately  4 | Severely  5 |
|  | Suffer in my personal relationships | Not at all  1 | Very mildly  2 | Mildly  3 | Moderately  4 | Severely  5 |
|  | Experience trouble for food and housing | Not at all  1 | Very mildly  2 | Mildly  3 | Moderately  4 | Severely  5 |
